# *Lactobacillus casei* Ghosts as a Vehicle for the Delivery of DNA Vaccines Mediate Immune Responses

**DOI:** 10.3389/fimmu.2022.849409

**Published:** 2022-05-31

**Authors:** Xiaoli Yu, Li Wang, Xinru Yang, Songsong Zhang, Guiwei Li, Lanlan Zhang, Jiaxuan Li, Xiaona Wang, Han Zhou, Yanping Jiang, Wen Cui, Yijing Li, Lijie Tang, Xinyuan Qiao

**Affiliations:** ^1^ Heilongjiang Key Laboratory for Animal Disease Control and Pharmaceutical Development, Department of Preventive Veterinary Medicine, College of Veterinary Medicine, Northeast Agricultural University, Harbin, China; ^2^ Branch of Animal Husbandry and Veterinary, Heilongjiang Academy of Agricultural Sciences, Qiqihar, China; ^3^ Heilongjiang Fishery Technology Extension Station , Harbin, China

**Keywords:** delivery system, bacterial ghost, dendritic cell, macrophages, deoxyribonucleic acid (DNA) vaccines

## Abstract

We developed *Lactobacillus casei* bacterial ghosts (BGs) as vehicles for delivering DNA vaccines and analyzed their effects on immune responses. Uptake of the plasmids encoding the enhanced green fluorescent protein (pCI-EGFP) and BGs loaded with pCI-EGFP by macrophages was investigated using fluorescence microscopy and flow cytometry. The results showed that pCI-EGFP-loaded *L. casei* BGs were efficiently taken up by macrophages. *Lactobacillus casei* BGs loaded with plasmids encoding VP6 protein of PoRV (pCI-PoRV-VP6) significantly upregulated the mRNA expression of interleukin (IL)-1β, IL-10, tumor necrosis factor (TNF)-α, inducible nitric oxide synthase (iNOS), arginase-1 (Arg-1), Mannose receptor (CD206) toll-like receptor (TLR)-2, TLR4, and TLR9 in macrophages. The levels of markers of M1 polarization (IL-10 and TNF-α) and M2 polarization (Arg-1 and CD206) were increased in macrophages incubated with pCI-PoRV-VP6-loaded BGs compared with the control group. The results of the enzyme-linked immunosorbent assay showed that the secretion of IL-1β, IL-10, and TNF-α in macrophages was significantly upregulated compared with the control group. Flow cytometry demonstrated that *L. casei* BGs loaded with pCI-PoRV-VP6 promoted the maturation of dendritic cells (DCs). Following incubation with pCI-PoRV-VP6-loaded BGs, the mRNA expression levels of IL-1β, IL-6 and interferon (IFN)-γ in DCs were significantly increased. ELISA assay showed the secretion of the IL-1β, IL-6, IFN-γ IL-10 and TNF-α in DCs were upregulated significantly. Thus, *L. casei* BGs promoted the maturation and activation of DCs. We analyzed the stimulatory capacity of DCs in a mixed lymphocyte reaction with allogeneic T cells. T cell proliferation increased upon incubation with DCs stimulated by BGs. After immunizing mice with BGs loaded with pCI-PoRV-VP6, the specific IgG levels in the serum were higher than those elicited by BGs loaded with pCI-PoRV-VP6. BGs loaded with pCI-PoRV-VP6 on Th1 and Th2 cytokines polarized T cells into the Th1 type and increased the proportion of CD4^+^/CD8^+^ T cells. These results indicate *L. casei* BGs effectively mediate immune responses and can be used as delivery system for DNA vaccination.

## Introduction

DNA vaccines are a powerful technology that has revolutionized vaccine research (1). These vaccines have many advantages over conventional vaccines. However, DNA vaccines show poor immunogenicity compared with traditional vaccines. Furthermore, they cannot effectively target antigen-presenting cells (APCs) and require high plasmid dosages (2, 3). Therefore, improving the immune response of DNA vaccines is a key factor determining the immune response.

Bacterial ghosts (BGs) bacterial shells without contents that preserve the entire surface structure of bacteria, including the outer membrane proteins, and show potential as vaccine candidates (4). In addition, BGs can be loaded with DNA, proteins, and drugs and exploited as delivery systems (5–7). Because BGs contain many Toll-like receptor (TLR) agonists, they can effectively target APCs and elicit potent immune responses against BG-delivered antigens (8–10). However, the use of BGs prepared from pathogenic bacteria poses safety concerns because the bacteria membranes cannot be completely split during the preparation of BGs, resulting in their inherent tendency to revert to pathogenicity. Moreover, lipopolysaccharide (LPS) and endotoxic effects in BG envelopes cannot be eliminated (11). Currently, all reported BGs have been prepared from pathogenic bacteria, and most are made of gram-negative bacteria (12, 13). Therefore, safe host bacteria must be used to develop BGs for delivery system applications.

Some studies showed that BGs can present antigens to regulate the polarization of T helper 1 (Th1) and T helper 2 (Th2) immune responses (14). Other studies demonstrated that BGs effectively stimulate macrophages and monocytes and polarize the immune response towards Th1 (15). Dendritic cells (DCs) are APCs that modulate T-cell responses. These cells can produce effective immune responses and establish immunological memory (16, 17). Mature DCs not only induce the activation and proliferation of T cells, but also play a role in the initiation of immunological tolerance (18, 19). Macrophages are APCs responsible for processing antigens and presenting them to antigen-specific T cells. Activated macrophages induce the expression of costimulatory molecules and cytokines that promote continued stimulation of T cells and the induction of adaptive immunity, linking innate and adaptive immunity (20).

Successful delivery of DNA to APCs requires a suitable delivery system and adequate antigen formulation. BGs have been widely used as an adjuvant and a delivery carrier for many bacterial and viral antigens (21, 22). In this study, we evaluated the ability of *Lactobacillus casei* BGs to deliver DNA vaccines *in vivo* and *in vitro*. We examined the efficacy of *L. casei* BGs in mediating transgene expression in macrophages, interaction of *L. casei* BGs with macrophages, and induction of cytokine expression. The interaction of DCs with *L. casei* BGs and their influence on DC maturation and cytokine production were further analyzed. These findings improve the understanding of the function of BGs in presenting DNA to APCs and initiation and regulation of immune responses. Materials and Methods.

### Ethics Statement

This trial was conducted in accordance with the requirements of the regulations governing laboratory animals and the Charter of the Ethics Committee for Laboratory Animals of the Northeast Agricultural University.

### Bacterial Strains and Plasmids

The RAW264.7 cell line was purchased from the China Center for Type Culture Collection (Wuhan, China). *Lactobacillus casei* ATCC 393 was kindly provided by the Netherlands NIZO Institute (Ede, Netherlands). The recombinant strain pPG-2-hocb/*L. casei* 393 (23) and plasmid pCI-PoRV-VP6 were constructed and stored in our laboratory. Total RNA was extracted from porcine Rubulavirus (PoRV) (JL94 strain). After reverse transcription, VP6 (encoding VP6 of PoRV) was amplified from the product by polymerase chain reaction (PCR) using the forward primer 5′-GCTTAGCATACCATGGAGGTTCTGTACTCA-3′ and reverse primer 5′- GTCGACTCACTTAATCAACATGCT-3′. The gene fragment was 1275 bp in length. The VP6 gene was cut with the restriction enzymes *Nhe* I and *Sal* I and inserted into the pCI-neo vector (Promega, Madison, WI, USA) according to the manufacturer’s instructions. Recombinant plasmids were transformed into *Escherichia coli* DH5α cells. The plasmids were extracted using Kits for rapid extraction of plasmid DNA (Sigma, St. Louis, MO, USA) and stored at -20°C.

### Animals

BALB/c mice (specific pathogen-free) were purchased from Changsheng Biotechnology Limited (Liaoning, China). All animal experiments were performed and animals were maintained according to the Ethical Committee for Animal Sciences of Heilongjiang Province and international recommendations for animal welfare.

### Preparation of Bacterial Ghosts

The recombinants were inoculated in 50 mL MRS medium containing10 μg/mL of chloramphenicol (Cm) and cultivated at 37°C for 12 h. 2% levulose was then added to the culture and cultivated at 37°C for 36 h. *L. casei* BGs were produced by expressing the phage-derived holin. PH of the culture was regulated and maintained ranging from 5.5 to 6.5 with 2 mol/L NaOH every 12 h. When OD_600_ of the culture did not decline, the lysed cells were collected by centrifugation at 5000× g for 20 min and treated with gentamycin (50 mg/mL) and streptomycin (100 mg/mL) to kill the surviving bacteria. Subsequently the BGs were washed twice with phosphate-buffered saline (PBS, pH 7.4) and resuspended in 5% sucrose solution. The BGs were lyophilized and stored at 4°C.

### Loading BGs With DNA Plasmid

Plasmid DNA was loaded into the BGs *via* diffusion through lysis holes. Lyophilized BGs (30 mg) were suspended in 10 mM sodium acetate, 100 mM NaCl, and 10 mM HEPES (pH 7.5) containing pET30a/PoRV/VP6 and pEGFP-C1 (final concentration 1 mg/mL). The samples were mixed with CaCl_2_ (final concentration, 25 mM) and agitated at 37°C for 15 min. Subsequently, the BGs were centrifuged at 10,000 ×*g* for 20 min; the pellets were washed once and resuspended in PBS.

### Transfection Experiments

BALB/c mice were administered Dulbecco’s Modified Eagle Medium (DMEM) by intraperitoneal injection and then the ascites fluid was extracted. Peritoneal macrophages in the ascites fluid were centrifuged at 2000 ×*g* for 20 min. Both peritoneal macrophages and RAW264.7 macrophages were cultured in DMEM containing 10% fetal bovine serum (Sigma, St. Louis, MO, USA). For transfection experiments, RAW264.7 macrophages and peritoneal macrophages were inoculated into 24-well plates (1 × 10^5^/well) and incubated with pCI-EGFP (1 mg/mL) and BGs loaded with pCI-EGFP (1000 bacteria/cell) for 2 h for attachment to and processing by the macrophages. The cells were washed with PBS to remove unabsorbed BGs and then incubated at 37°C for another 48 h. EGFP expression and transfection efficiency were determined by performing fluorescence microscopy and flow cytometry, respectively, with a FACS caliber (BD Biosciences, Franklin Lakes, NJ, USA). Untreated cells were used as controls.

### Real-Time PCR Assay of Marker Expression by Macrophages

RAW264.7 macrophages and peritoneal macrophages were inoculated into 24-well plates (1 × 10^5^/well) and cultured at 37°C for 12 h. The cells were incubated with plasmid pCI-PoRV-VP6 (1 mg/mL), BGs (1000 bacteria/cell), and BGs loaded with pCI-PoRV-VP6 (1000 bacteria/cell) at 37°C for 2 h. After washing three times with PBS, the cells were cultured in fresh medium at 37°C. Total RNA was extracted after cultivation for 6, 12, 18, 24, and 30 h using an RNeasy Mini Kit (Feijie, Shanghai, China) according to the manufacturer’s instructions. Reverse transcription was performed; the A280/A260 ratio of the RNA was 1.8–2.0 and thus the RNA could be used to prepare cDNA. cDNA was synthesized from 1.0 μg of RNA using a transcriptase kit (Takara, Dalian, China) according to the manufacturer’s instructions. The mRNA levels of interleukin (IL)-1β, IL-10, inducible nitric oxide synthase (iNOS), arginase-1 (Arg-1), mannose receptor (CD206), and tumor necrosis factor (TNF)-α were quantified using real-time PCR. The primers used to amplify the target genes are listed in **Supplementary Table S1**. To analyze gene expression, cDNA was amplified using Power SYBR Green PCR Master Mix (Promega) (24). Each gene was amplified in triplicate. The expression levels of cytokine mRNA (normalized to that of GAPDH) were measured using the 2^-ΔΔCT^ method (24).

### ELISA of Cytokine Secretion in Macrophages

RAW264.7 macrophages and peritoneal macrophages were inoculated into 24-well plates (1 × 10^5^/well) and cultured at 37°C for 12 h. Subsequently, the cells were incubated with plasmid pCI-PoRV-VP6 (1 mg/mL), BGs (1000 bacteria/cell), and pCI-PoRV-VP6-loaded BGs (1000 bacteria/cell) at 37°C for 2 h. After washing three times with PBS, the cells were cultured in fresh medium at 37°C. After cultivation for 6, 12, 18, 24, and 30 h, the IL-1β, IL-10, and TNF-α levels in the supernatant were detected using an enzyme-linked immunosorbent assay (ELISA) kit (Meimian, Jiangsu, China) according to the manufacturer’s instructions.

### Preparation of DCs Derived From Bone Marrow Cells

DCs derived from bone marrow cells were obtained from the femurs of mice and harvested by density gradient centrifugation (24, 25). Interphase cells were isolated and washed three times with PBS. The DCs were cultured in 6-well plates (5 × 10^6^ cells/well) in RPMI-1640 medium (Sigma) containing 10% fetal calf serum (PeproTech, Rocky Hill, NJ, USA), streptomycin (100 μg/mL) (PeproTech), 40 ng/mL granulocyte-macrophage colony-stimulating factor (PeproTech), and 20 ng/mL IL-4 (PeproTech). Unattached cells were removed by washing after 3 h, and the attached cells were cultured. The culture medium was replaced with fresh medium containing the same concentration of cytokines every two days. After 7 days, nonadherent cells were collected, inoculated into six-well plates, and cultured in incomplete RPMI-1640 medium supplemented with IL-4 (10 ng/mL) and granulocyte-macrophage colony-stimulating factor (20 ng/mL) for another 2 days. The DCs were harvested for further experiments.

### Flow Cytometry Assay of Marker Expression by Mature DCs

For stimulation experiments, DCs (1 × 10^6^/mL) were inoculated into six-well plates with RPMI-1640 medium and incubated with pCI-PoRV-VP6 (1 mg/mL), BGs loaded with pCI-PoRV-VP6 (1000 bacteria/cell), or LPS (1 μg/mL) for 24 h. The treated DCs were collected and washed twice with staining buffer (0.1% NaN_3_ and 0.5% fetal calf serum in PBS), followed by incubation with fluorescein isothiocyanate-conjugated anti-mouse major histocompatibility complex (MHC) class II, CD80, and CD86 antibodies (Sigma, St. Louis, MO, USA) at 4°C for 30 min. Fluorescein isothiocyanate-conjugated isotype control antibodies were used as negative controls. The cells were analyzed using a FACS Caliber. The results were analyzed using BD FACSDIVA software (BD Biosciences).

### Real-Time PCR Assay of Marker Expression by DCs

DC cultures stimulated with pCI-PoRV-VP6 (1 mg/mL), BGs loaded with pCI-PoRV-VP6 (1000 bacteria/cell), or LPS (1 μg/mL) for 24 h were harvested. Total RNA was extracted as described in 2.7. The mRNA levels of IL-1β, IL-6, IFN-γ IL-10 and TNF-α were quantified using quantitative reverse transcription PCR (RT-qPCR). The primers used for the target genes are listed in Table 1. To analyze gene expression, cDNA was amplified using Power SYBR Green PCR Master Mix (24). Each gene was amplified in triplicate, and the levels of cytokine mRNA (normalized to that of GAPDH) were analyzed using the 2^-ΔΔCT^ method as described previously (24).

### ELISA of Cytokine Secretion by DCs

Cytokine secretion by DCs was analyzed using ELISA. DC cultures stimulated with pCI-PoRV-VP6 (1 mg/mL), pCI-PoRV-VP6-loaded BGs (1000 bacteria/cell), or LPS (1 μg/mL) for 24 h were harvested. The levels of IL-1β, IL-6, IFN-γ IL-10 and TNF-α in the cell supernatant were detected using an ELISA kit (Meimian, Jiangsu, China) according to the manufacturer’s instructions.

### Mixed Leukocyte Reaction

DCs were cultured for 7 days and then incubated with pCI-PoRV-VP6 (1 mg/mL), pCI-PoRV-VP6 loaded with BGs (1000 particles/cell), or LPS (100 ng/well) for 24 h. Mitomycin C (50 mg/mL) (Merck KgaA, Darmstadt, Germany) was added to the cultured DCs and then incubated for 2 h at 37°C. The treated DCs were washed three times with PBS. T lymphocytes (1 × 10^5^/mL) were cultured in triplicate in 96-well culture plates and then mixed with cultured DCs at ratios of 1:1, 1:10, and 1:100. Splenocytes stimulated by ConA (5 µg/mL) were used as positive controls, splenocytes were used as negative controls, and 1640 culture medium was used as a blank control. Mixed cells were cultured for 72 h at 37°C in 96-well plates. Untreated T-cells served as negative controls. T cells proliferation was analyzed using the methylthiazolyldiphenyl-tetrazolium bromide (MTT) method (26). The stimulation index (SI) was calculated as the mean absorbance by subtracting the OD_600_ from the OD_570_ after subtracting the absorbance reading for untreated cells at each wavelength.

### Immunization

Groups of eight-week-old BALB/c mice (n = 5) were immunized on days 0, 14, and 28 *via* the oral, intramuscular injection, or intraperitoneal injection route with pCI-PoRV-VP6 alone (80 μg), BGs loaded with pCI-PoRV-VP6 (10^9^ colony-forming units, containing 80 μg of DNA), unloaded BGs, and PBS, respectively. Serum samples were collected from the mice on days 1, 7, 14, 21, 28, 35, and 42 after immunization and stored at -20°C until ELISA.

### ELISA of IgG in Serum

Specific antibodies in the serum were analyzed by using ELISA. VP6 protein was coated at 2 μg per well in polystyrene microtiter plates at 4°C for 12 h (purified VP6 protein expressed in *E. coli* was prepared in our laboratory). The ELISA plates were washed with PBS containing 1% Tween-20 three times and then blocked with PBS containing 5% skim milk at 37°C for 3 h. The serum was serially diluted and incubated at 37°C for 1 h after washing three times. The samples were incubated with horseradish peroxidase-conjugated goat anti-mouse IgG (Sigma) diluted at 1:3000, washed, and mixed with 100 μL of *o*-phenylene diamine dihydrochloride substrate (Sigma). The OD_490_ was measured.

### Lymphoproliferation Assay

Mice from each group were sacrificed at 14 days after the last immunization, and their spleens were removed aseptically. The spleens were minced and then pressed through a fine copper wire screen mesh (100 μm). The sample was collected by centrifugation 456 g for 10 min, followed by incubation in 15 mL of 0.7% NH_4_Cl at 37°C for 10 min. The liquid was then centrifuged at 465 g for 10 min. The deposit was washed three times with RPMI-1640 medium after discarding the supernatant. The splenocytes were diluted to 1 × 10^6^/mL in RPM-I1640 medium containing 10% fetal bovine serum and then co-cultured with VP6 protein (0.5 or 5 μg/mL) at 37°C for 72 h. Splenocytes stimulated with ConA (5 μg/mL) and untreated cells served as positive and negative controls, respectively. Cell proliferation was measured in an MTT assay (26). The SI was calculated as the mean absorbance by subtracting the OD_600_ from the OD_570_ after subtracting the absorbance reading for untreated cells at each wavelength.

### Flow Cytometry Analysis of CD4^+^ and CD8^+^ T Cells

Spleen lymphocytes from each group were prepared as described in 2.16. Splenocytes were diluted to 1 × 10^6^/mL and incubated with antibodies against anti-CD3-fluorescein isothiocyanate, anti-CD4-biotin (BIOT, Hangzhou, China), and anti-CD8-phycoerythrin (Sigma) in the dark at 4°C for 30 min. After washing three times with PBS, all samples were analyzed by flow cytometry using a FACS Caliber. The results were analyzed using BD FACSDIVA software.

### Th1 and Th2 Cytokine Analysis

Serum samples were collected at 24 h after immunization. Th1 cytokine (IFN-γ) and Th2 cytokine (IL-4) levels were analyzed using an ELISA kit (Meimian, China) according to the manufacturer’s instructions.

### Statistical Analysis

The data are expressed as the mean ± standard deviation (SD). Statistical data were analyzed using SPSS software (SPSS, Inc., Chicago, IL, USA) for analysis of variance.^*^P < 0.05, ^**^P < 0.01, ^***^P < 0.001; ^a^P < 0.05, ^b^P < 0.01, ^c^P < 0.001; ^#^P < 0.05, ^##^P < 0.01, ^###^P < 0.001. were considered to indicate statistically significant differences.

## Results

### EGFP Expression in Macrophages by Ghost-Mediated Transfection

**Figure 1:**
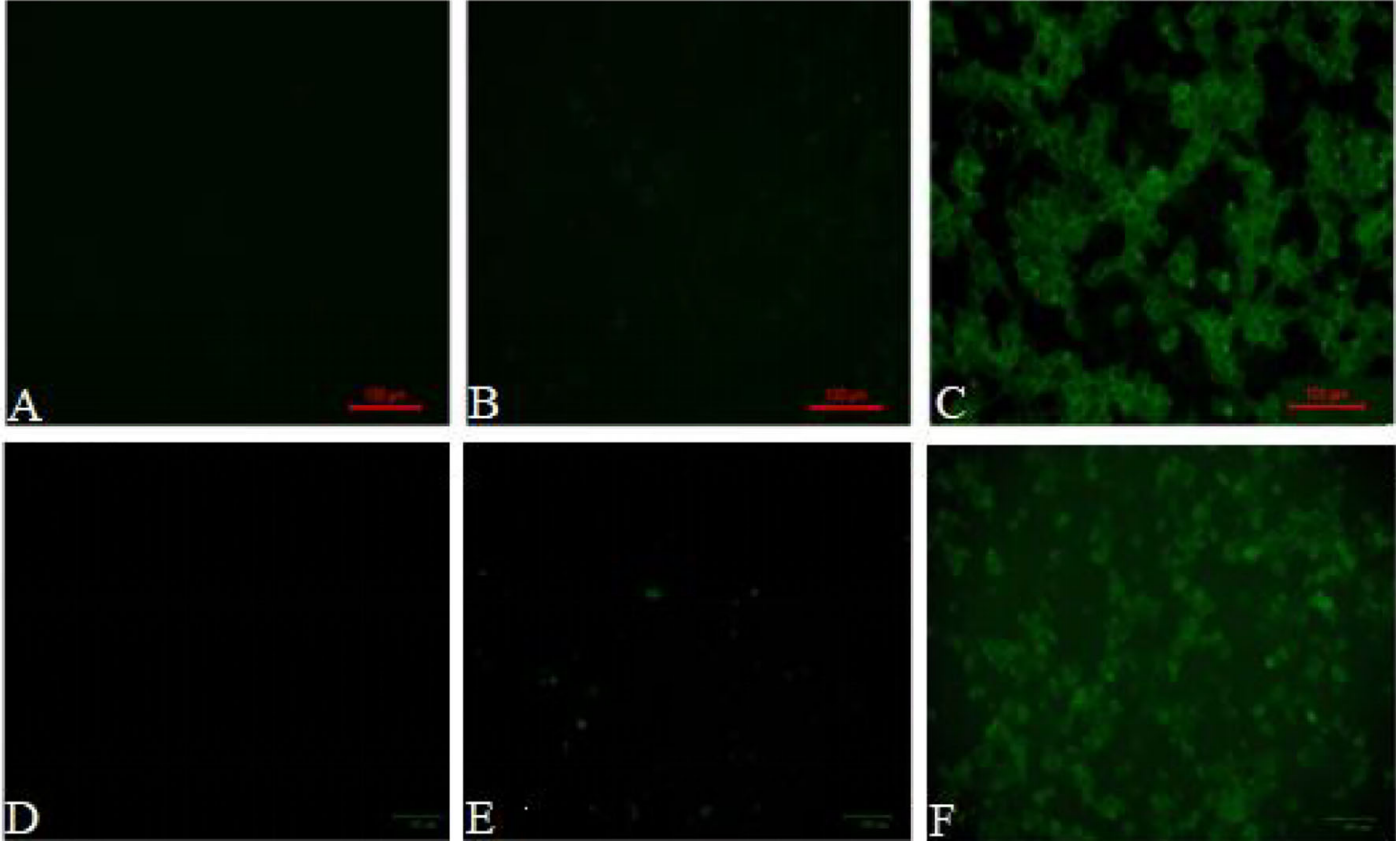
EGFP expression in in RAW264.7 cells and peritoneal macrophages was examined by fluorescence microscopy. Fluorescence microphotograph of **(A)** untreated RAW264.7 cells, **(B)** RAW264.7 cells incubated with pCI–EGFP (1 mg/mL), **(C)** RAW264.7 cells incubated with pCI–EGFP–loaded BGs, **(D)** untreated peritoneal macrophages, **(E)** peritoneal macrophages incubated with pCI–EGFP (1 mg/mL), and **(F)** peritoneal macrophages incubated with pCI–EGFP–loaded BGs.

**Figure 2:**
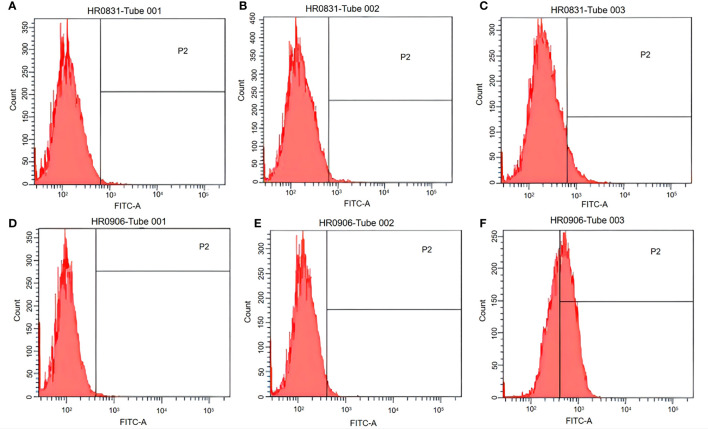
EGFP expression in RAW264.7 cells and peritoneal macrophages was examined using flow cytometry. **(A)** Untreated RAW264.7 cells. **(B)** RAW264.7 cells incubated with pCI–EGFP (1 mg/mL). **(C)** RAW264.7 cells incubated with pCI–EGFP–loaded bacterial ghosts (BGs). **(D)** Untreated peritoneal macrophages. **(E)** Peritoneal macrophages incubated with pCI–EGFP (1 mg/mL). **(F)** Peritoneal macrophages incubated with pCI–EGFP–loaded BGs.

Both peritoneal macrophages and RAW264.7 cells were incubated with pCI-EGFP and pCI-EGFP-loaded BGs, respectively. Uptake of pCI-EGFP and BGs loaded with pCI-EGFP by macrophages was investigated using fluorescence microscopy (**Figure 1**) and flow cytometry (**Figure 2**). Most RAW264.7 cells (**Figure 1C**) and peritoneal macrophages (**Figure 1F**) exhibited green fluorescence in their cytoplasm after incubation with pCI-EGFP-loaded BGs. Only a small amount of green fluorescence was observed in RAW264.7 cells (**Figure 1E**) and peritoneal macrophages (**Figure 1B**) incubated with pCI-EGFP. No green fluorescence was observed in untreated cells (**Figures 1A, D**). We further investigated EGFP expression in the macrophages using flow cytometry. The fluorescence observed by flow cytometry in RAW264.7 and peritoneal macrophages was increased from 1.4% to 7.2% (**Figures 2B, C**) and 1.8% to 57.6% (**Figures 2E, F**), respectively, confirming the results of fluorescence microscopy. These results suggest that pCI-EGFP-loaded *L. casei* BGs can be taken up more efficiently by macrophages. The more efficient expression of EGFP was mediated by *L. casei* BGs compared to in control cells.

### BGs Regulate mRNA Expression of Cytokines and TLRs in Macrophages

**Figure 3:**
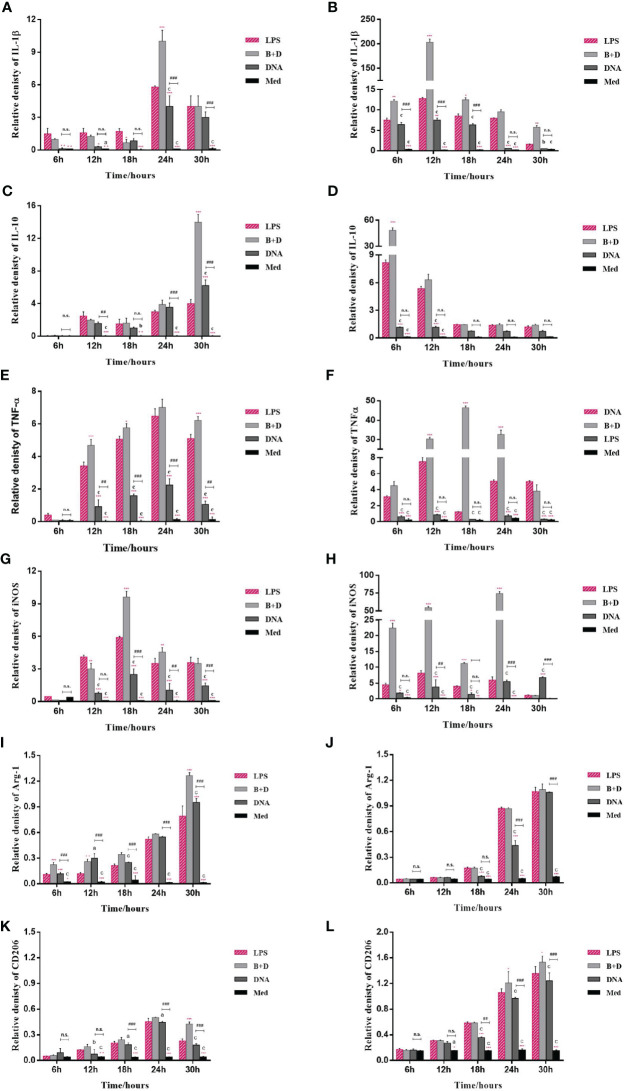
mRNA expression of inflammatory factors in RAW264.7 cells and peritoneal macrophages. RAW264.7 cells and peritoneal macrophages were incubated with plasmid pCI–PoRV–VP6 (1 mg/mL) and bacterial ghosts (BGs) loaded with pCI–PoRV–VP6 (1000 bacteria/cell), respectively. Total RNA was extracted at different times, and the mRNA expression of inflammatory factors was quantified using RT–qPCR. mRNA expression of **(A)** IL–1β in RAW264.7 cells, **(B)** IL–1β in peritoneal macrophages, **(C)** IL–10 in RAW264.7 cells, **(D)** IL–10 in peritoneal macrophages, **(E)** TNF–α in RAW264.7 cells, **(F)** TNF–α in peritoneal macrophages, **(G)** iNOS in RAW264.7 cells, **(H)** iNOS in peritoneal macrophages, **(I)** Arg–1 in RAW264.7 cells, **(J)** Arg–1 in peritoneal macrophages, **(K)** CD206 in RAW264.7 cells, and **(L)** CD206 in peritoneal macrophages. Values are expressed as the mean ± SD of four individual experiments. Significance of differences between the LPS, BG+DNA, DNA, and Macrophage culture medium (Med) groups is represented as follows: ^*^P < 0.05, ^**^P < 0.01, ^***^P < 0.001. Letter indicates a significant differences between BG+DNA, DNA, and Med groups: ^a^P < 0.05, ^b^P < 0.01, ^c^P < 0.001. #Asterisk indicates significant difference between DNA groups and Med groups: ^#^P < 0.05, ^##^P < 0.01, ^###^P < 0.001. n.s., no significance.

**Figure 4:**
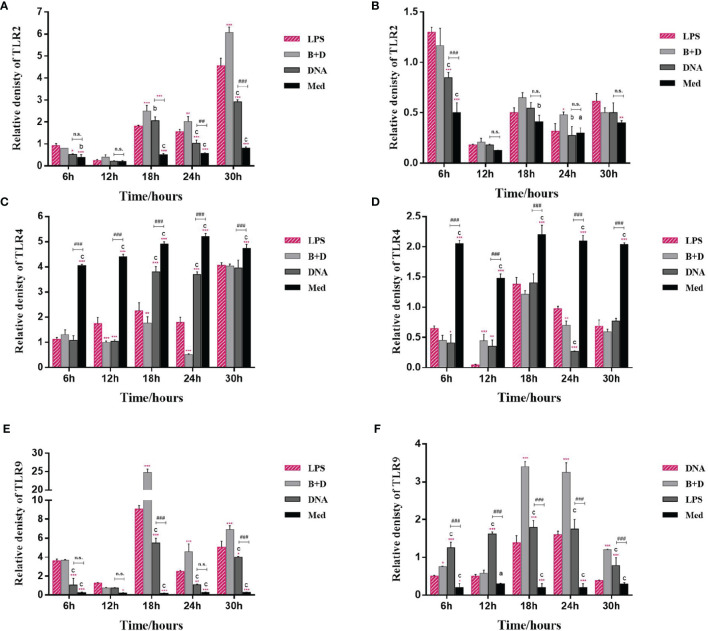
mRNA expression of TLRs in macrophages. RAW264.7 cells and peritoneal macrophages were incubated with plasmid pCI–PoRV–VP6 (1 mg/mL) and bacterial ghosts (BGs) loaded with pCI–PoRV–VP6 (1000 bacteria/cell), respectively, at 37°C for 2 h. Total RNA was extracted at different times. The mRNA levels expression of TLRs were quantified using RT–qPCR. mRNA expression of **(A)** TLR2 in RAW264.7 cells, **(B)** TLR2 in peritoneal macrophages, **(C)** TLR4 in RAW264.7 cells, **(D)** TLR4 in peritoneal macrophages, **(E)** TLR9 in RAW264.7 cells, and **(F)** TLR9 in peritoneal macrophages. Values are expressed as the mean ± SD of four individual experiments. Significance of differences between the LPS, BG+DNA, DNA, and Med groups is represented as follows: ^*^P < 0.05, ^**^P < 0.01, ^***^P < 0.001. Letter indicates significant differences between BG+DNA, DNA, and Med groups: ^a^P < 0.05, ^b^P < 0.01, ^c^P < 0.001. #Asterisk indicates a significant difference between DNA and Med groups: ^#^P < 0.05, ^##^P < 0.01, ^###^P < 0.001. n.s., no significance.

The potential of BGs to regulate the mRNA expression of TLRs and cytokines in macrophages was investigated using RT-qPCR. The RAW264.7 cells incubated with pCI-PoRV-VP6-loaded BGs expressed higher levels of IL-1β, IL-10, TNF-α, iNOS, TLR2, and TLR9 compared to cells incubated with pCI-PoRV-VP6 (**Figures 3**, **4**). The expression of IL-1β, IL-10, TNF-α, iNOS, TLR2, TLR4, and TLR9 peaked at 24, 30, 24, 18, 30, 30, and 18 h, respectively (**Figures 3A, C, F, G**and **Figures 4A, C, E**). After incubation with pCI-PoRV-VP6-loaded BGs, the mRNA expression levels of IL-1β, IL-10, TNF-α, iNOS, TLR2, and TLR9 in peritoneal macrophages were significantly increased. The expression of IL-1β, IL-10, TNF-α, iNOS, TLR2, TLR4, and TLR9 in peritoneal macrophages peaked at 12, 6, 12, 24, 6, 18, and 18 h, respectively (**Figures 3B, D, F, H** and **Figures 4B, D, H**). These results demonstrate that *L. casei* BGs significantly upregulated the mRNA expression levels of most inflammatory cytokines and TLRs in macrophages. The expression levels of different inflammatory cytokines and TLRs peaked at different times.

The markers of M1 polarization (**Figures 3C–F**) and M2 polarization (**Figures 3G, I, K, L**) were all higher in macrophages incubated with pCI-PoRV-VP6-loaded BGs compared to in control cells. These results indicate that both M1-type and M2-type polarization was induced by BGs, with greater effects on M1-type polarization.

### Analysis of Cytokines in Macrophages by ELISA

**Figure 5:**
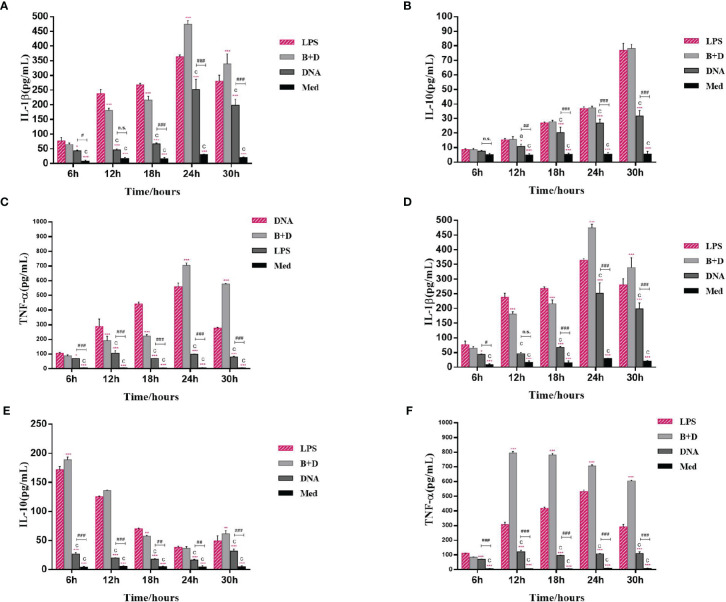
Levels of IL–1β, IL–10, and TNF–α secretion from RAW264.7 cells and peritoneal macrophages. RAW264.7 cells and peritoneal macrophages were incubated with plasmid pCI–PoRV–VP6 (1 mg/mL) and bacterial ghosts (BGs) loaded with pCI–PoRV–VP6 (1000 bacteria/cell), respectively, at 37°C for 2 h. After cultivation for 6, 12, 18, 24 and 30 h, the IL–1β, IL–10, and TNF–α contents in the cell supernatant were detected using ELISA. Levels of **(A)** IL–1β secretion from RAW264.7 cells, **(B)** IL–10 secretion from RAW264.7 cells, **(C)** TNF–α secretion from RAW264.7 cells, **(D)** IL–1β secretion from peritoneal macrophages, **(E)** IL–10 secretion from peritoneal macrophages, and **(F)** TNF–α secretion from peritoneal macrophages. Values are expressed as the mean ± SD of four individual experiments. Significance of differences between the LPS, BG+DNA, DNA, and Med groups is represented as follows: ^*^P < 0.05, ^**^P < 0.01, ^***^P < 0.001. Letter indicates significant differences between BG+DNA, DNA, and Med groups: ^a^P < 0.05, ^b^P < 0.01, ^c^P < 0.001. #Asterisk indicates a significant difference between DNA and Med groups: ^#^P < 0.05, ^##^P < 0.01, ^###^P < 0.001. n.s., no significance.

Cytokine secretion by macrophages was analyzed using ELISA. After incubation with pCI-PoRV-VP6-loaded BGs, the secretion of IL-1β, IL-10, and TNF-α in RAW264.7 cells peaked at 24, 30, and 24 h, respectively (**Figures 5A–C**). The secretion of IL-1β, IL-10, and TNF-α in peritoneal macrophages peaked at 24, 6, and 12 h, respectively (**Figures 5D–F**). These results show that *L. casei* BGs significantly upregulated the levels of most of the evaluated inflammatory cytokines in macrophages.

### *Lactobacillus casei* BGs Promote DC Maturation

**Figure 6:**
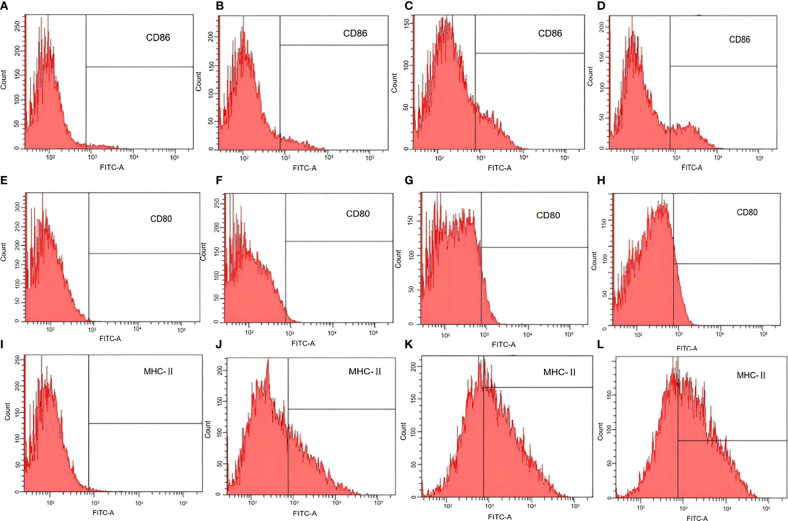
Flow cytometry assay of marker expression by mature dendritic cells (DCs). DCs were treated with plasmid DNA (1 mg/mL), BGs loaded with plasmid DNA (1000 bacteria/cell), or lipopolysaccharide (LPS, 1 μg/mL). MHC class II, CD80, and CD86 in mature DCs were analyzed using flow cytometry. Expression of **(A)** CD86 in untreated DCs, **(B)** CD86 in DCs treated with plasmid DNA, **(C)** CD86 in DCs treated with plasmid DNA–loaded BGs, **(D)** CD86 in DCs treated with LPS, **(E)** CD80 in untreated DCs, **(F)** CD80 in DCs treated with plasmid DNA, **(G)** CD80 in DCs treated with plasmid DNA–loaded BGs, **(H)** CD80 in DCs treated with LPS, **(I)** MHC–II in untreated DCs, **(J)** MHC–II in DCs treated with plasmid DNA, **(K)** MHC–II in DCs treated with plasmid DNA–loaded BGs, and **(L)** MHC–II in DCs treated with LPS.

The antigen-presenting co-stimulatory molecules CD80, CD86, and MHC class II are surface markers of mature DCs. To analyze the influence of *L. casei* BGs on the maturation of bone marrow derived DCs, CD80, CD86, and MHC class II expression in DCs was analyzed using flow cytometry. In untreated DCs, the positivity rates of CD86, CD80, and MHC-II were 3.2% (**Figure 6A**), 0% (**Figure 6E**), and 1% (**Figure 6I**), respectively. After pCI-PoRV-VP6 stimulation of DCs, the positive rates of CD86, CD80, and MHC-II were 3.7% (**Figure 6B**), 1.5% (**Figure 6F**), and 37.4% (**Figure 6J**), respectively. After the DCs were stimulated by DNA-packed *L. casei* BGs, the positive rates of CD86, CD80, and MHC-II were 22.4% (**Figure 6C**), 12.0% (**Figure 6G**), and 60.5% (**Figure 6K**), respectively. In DCs treated with LPS, the positivity rates of CD86, CD80, and MHC-II were 19.8% (**Figure 6D**), 7.0% (**Figure 6H**), and 42.1% (**Figure 6L**), respectively. Preincubation with DNA-loaded BGs for 24 h led to increased expression of MHC class II in DCs treated with DNA (**Figures 6J, K**). The expression of CD80 and CD86 was also upregulated after treatment with DNA-loaded *L. casei* BGs compared to that in DCs treated with DNA (**Figures 6B, C, F, G**). Stimulation with *L. casei* BGs significantly enhanced MHC-II, CD80, and CD86 expression, indicating that *L. casei* BGs promoted DC maturation.

### *Lactobacillus casei* BGs Induce Cytokine mRNA Expression in DCs

**Figure 7:**
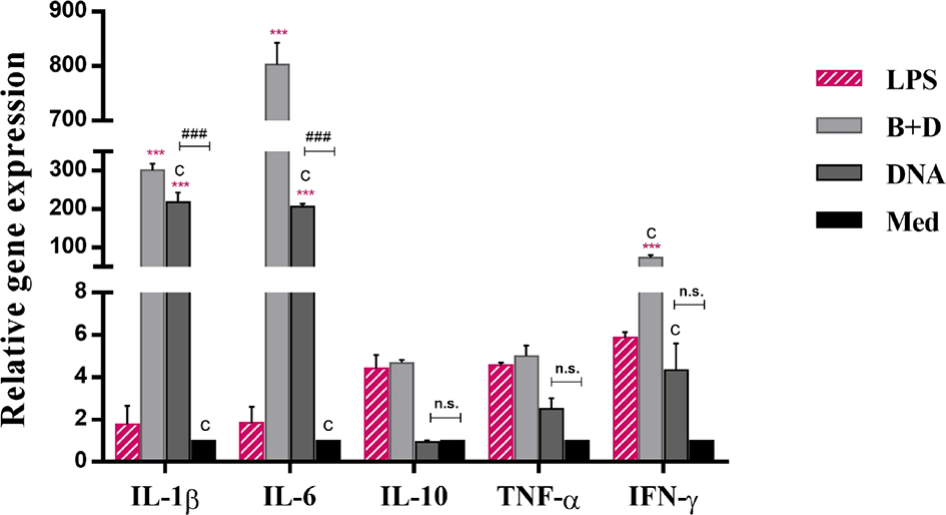
Expression of IL–1β, IL–6, IL–10, TNF–α, and IFN–γ mRNA levels in dendritic cells (DCs). DCs were incubated with pCI–PoRV–VP6 plasmid DNA (1 mg/mL) and bacterial ghosts (BGs) loaded with pCI–PoRV–VP6 plasmid DNA (1000 bacteria/cell) respectively at 37°C for 24 h. Total RNA was extracted. IL–1β, IL–6, IL–10, TNF–α, and IFN–γ mRNA levels were quantified using RT–qPCR. Values are expressed as the mean ± SD of four individual experiments. Significance of differences between the LPS, BG+DNA, DNA, and Med groups are represented as follows: ^*^P < 0.05, ^* *^P < 0.01, ^***^P < 0.001. Letter indicates significant differences between BG+DNA, DNA, and Med groups: ^a^P < 0.05, ^b^P < 0.01, ^c^P < 0.001. #Asterisk indicates significant difference between DNA and Med groups: ^#^P < 0.05, ^##^P < 0.01, ^###^P < 0.001. n.s., no significance.

To analyze the effect of *L. casei* BGs on the activation of bone marrow-derived DCs, DCs were pre-incubated with DNA and DNA-loaded BGs for 24 h. The mRNA expression levels of TNF-α, IL-1β, IL-6, IL-10, and IFN-γ were investigated using RT-qPCR (**Figure 7**). Lower levels of inflammatory cytokines were observed in untreated cells. Following incubation with DNA-loaded BGs, the mRNA expression levels of IL-1β, IL-6 and IFN-γ inflammatory cytokines in DCs increased significantly compared to those in DCs incubated with DNA. The mRNA expression of IL-10 and TNF-α were also increased, but it was inapparent compared to those in the DCs incubated with DNA. These results showed that L. casei BGs upregulated the levels of most of these inflammatory cytokines in DCs significantly.

### Analysis of Cytokines in DCs by ELISA

**Figure 8:**
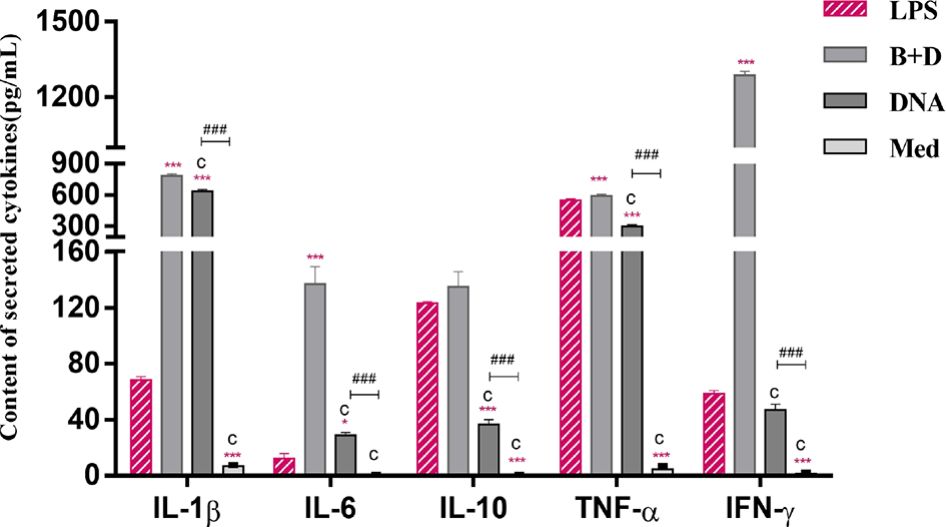
Secretion of IL–1β, IL–6, IL–10, TNF–α and IFN–γ levels in dendritic cells (DCs). DCs were incubated with pCI–PoRV–VP6 plasmid DNA (1 mg/mL) and bacterial ghosts (BGs) loaded with pCI–PoRV–VP6 plasmid DNA (1000 bacteria/cell) at 37°C for 24 h. Cytokine contents by DCs were analyzed using ELISA. Values are expressed as the mean ± SD of four individual experiments. Significance of differences between the LPS, BG+DNA, DNA, and Med groups is represented as follows: ^*^P < 0.05, ^**^P < 0.01, ^***^P < 0.001. Letter indicates significant differences between BG +DNA groups, DNA and Med groups: ^a^P < 0.05, ^b^P < 0.01, ^c^P < 0.001. #Asterisk indicate significant difference between DNA and Med groups: ^#^P < 0.05, ^##^P < 0.01, ^###^P < 0.001.

The cytokine secretion levels of TNF-α, IL-1β, IL-6, IL-10, and IFN-γ in DCs were analyzed using ELISA. Following incubation with DNA-loaded BGs, the secretion levels of all inflammatory cytokines in DCs were increased significantly compared to those in DCs incubated with DNA (**Figure 8**). These results demonstrate that incubation of immature DCs with DNA-loaded *L. casei* BGs resulted in the maturation and activation of DCs. Thus, *L. casei* BGs significantly upregulated the levels of most of inflammatory cytokines in DCs. These results demonstrate that incubation of immature DCs with DNA-loaded *L. casei* BGs resulted in the maturation and activation of DCs. *Lactobacillus casei* BGs activated DCs to produce both anti-inflammatory and proinflammatory cytokines, which may improve immune responses using *L. casei* BGs as a delivery system for DNA vaccines.

### *Lactobacillus casei* BGs Increase Allostimulatory Capacity of DCs

**Figure 9:**
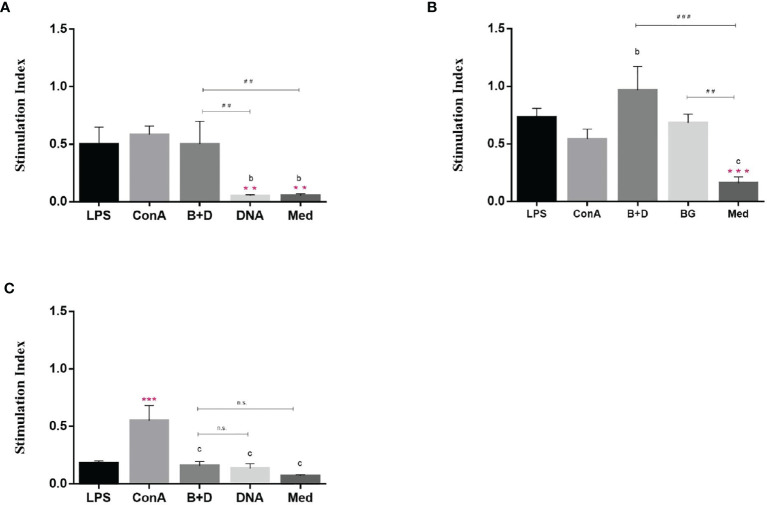
Stimulatory effect of dendritic cells (DCs) by mixed lymphocyte reaction. DCs were stimulated with lipopolysaccharide (LPS, 100 ng/well), ConA (0.5 ng/well), plasmid DNA (1 mg/mL), or DNA–loaded bacterial ghosts (BGs, 1000 particles/cell) for 24 h. T lymphocytes were cultured in triplicate in 96–well culture plates and then mixed with the cultured DCs at ratios of 1:1 **(A)**, 10:1 **(B)** and 100:1 **(C)**. T cell proliferation was measured using an MTT assay, the results are expressed as the stimulation index (SI). Values are expressed as the mean ± SD of five individual experiments. Significance of differences between the LPS, ConA, BG+DNA, DNA, and Med groups is represented as follows: ^*^P < 0.05, ^**^P < 0.01, ^***^P < 0.001. Letter indicates significant differences between ConA, BG+DNA, DNA, and Med groups: ^a^P < 0.05, ^b^P < 0.01, ^c^P < 0.001. #Asterisk indicates significant difference between, BG+DNA, DNA, and Med groups: ^#^P < 0.05, ^##^P < 0.01, ^###^P < 0.001. n.s., no significance.

A remarkable property of DCs is their ability to stimulate naïve T cells and present antigens *in vivo*. This ability can be analyzed *in vitro* in a mixed leukocyte reaction. DCs were cultured for 7 days and then incubated with LPS, ConA, plasmid DNA, or plasmid DNA-loaded BGs for 24 h. We then analyzed the stimulatory capacity of DCs in the mixed leukocyte reaction with allogeneic T cells. DCs treated with DNA-loaded BGs or LPS significantly activated allogenic T cells at APC: T ratios of 1:1 and 10:1 (**Figure 9**) compared to in DCs treated with DNA. The results showed that T cell proliferation responses increased when DCs were stimulated with BGs. Thus, the effect of DCs on maturing T cells was enhanced following incubation with DNA-loaded BGs. Mature DCs stimulate the activation and proliferation of naïve T cells *in vivo*. Our findings demonstrate that *L. casei* BGs efficiently promoted the maturation of DCs and improved the allostimulatory capacity of DCs.

### Determination of Specific IgG Antibody

**Figure 10:**
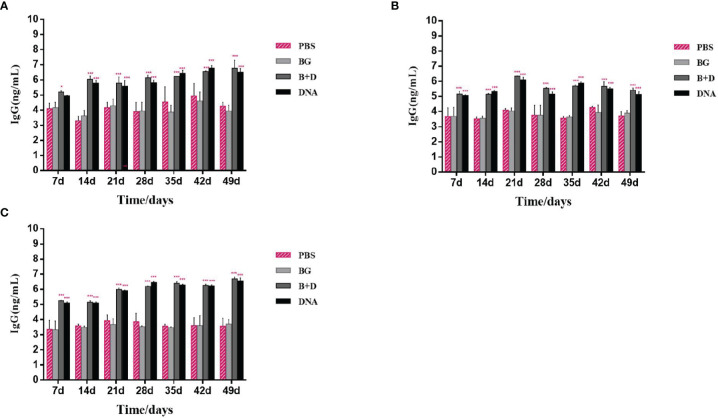
Immunoglobulin antibodies in serum. Groups of mice were immunized **(A)** orally, **(B)** intramuscularly, or **(C)** intraperitoneally with pCI–PoRV–VP6, bacterial ghosts (BGs) loaded with pCI–PoRV–VP6, unloaded BGs, and PBS. Serum samples were collected on days 0, 7, 14, 21, 27–29, and 49. Values are expressed as the mean ± SD of four individual experiments. Significance of differences between the PBS, BG+DNA, DNA, and BG groups is represented as follows: ^*^P < 0.05, ^**^P < 0.01, ^***^P < 0.001. n.s., no significance.

Groups of mice were immunized orally (**Figure 10A**), intramuscularly (**Figure 10B**), or intraperitoneally (**Figure 10C**) with pCI-PoRV-VP6, BGs loaded with pCI-PoRV-VP6, unloaded BGs, and PBS, respectively. Serum samples were collected on days 1, 7, 14, 21, 28, 35, 42, and 49. Significant levels of specific anti-VP6 IgG were observed after immunization with pCI-PoRV-VP6 or BGs loaded with pCI-PoRV-VP6 compared to the group immunized with PBS or BGs (**Figures 10 A–C**). The specific IgG levels in serum samples elicited by DNA-loaded BGs were significantly higher at most of time points than those elicited by BGs administered with pCI-PoRV-VP6 (**Figure 10**). Group of DNA-loaded BGs showed higher IgG levels than group of DNA at most of time points except for a minority of the time points, for example day 14.

### Lymphoproliferation Assay

**Figure 11:**
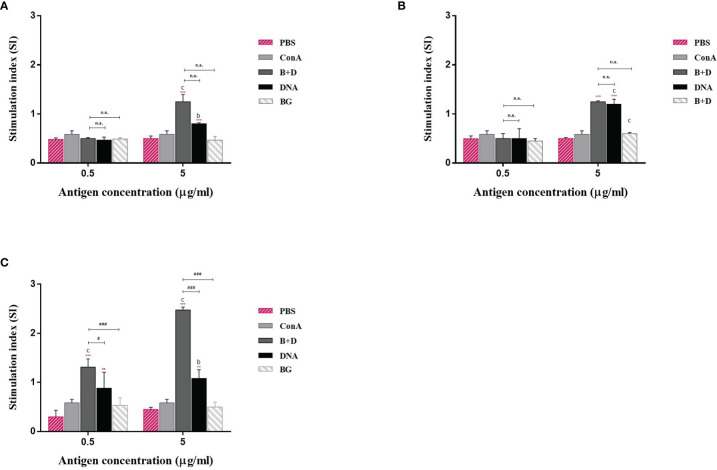
Lymphoproliferation assay. Lymphocytes from the mice immunized by the **(A)** oral, **(B)** intramuscular, or **(C)** intraperitoneal route were prepared 14 days after the last immunization and cultured with VP6 protein of final concentration of 0.5 or 5 μg/mL. The splenocytes stimulated with 5 μg/mL ConA as the positive control. The splenocytes untreated were used as negative controls. Values are expressed as the mean ± SD of five individual experiments. Significance of differences between the PBS, ConA, BG+DNA, DNA, and bacterial ghost (BG) groups is represented as follows: ^*^P < 0.05, ^**^P < 0.01, ^***^P < 0.001. Letter indicates significant differences between ConA, BG+DNA, DNA, and BG groups: ^a^P < 0.05, ^b^P < 0.01, ^c^P < 0.001. #Asterisk indicates significant difference between BG+DNA, DNA, and BG groups: ^#^P < 0.05, ^##^P < 0.01, ^###^P < 0.001.

Lymphocyte lymphoproliferation was examined to investigate the antigen-specific cell-mediated immune response. The lymphocytes of immunized mice proliferated **(Figure 11**). The stimulation index difference was significant in lymphocytes of the immunized mice incubated with VP6 protein at 5 μg/mL (**Figure 11**). No significant difference was observed in the group immunized *via* the oral or intramuscular route stimulated with VP6 protein at 5 μg/mL.

### Measurement of CD4^+^ and CD8^+^ T Cells in Immunized Mice

**Figure 12:**
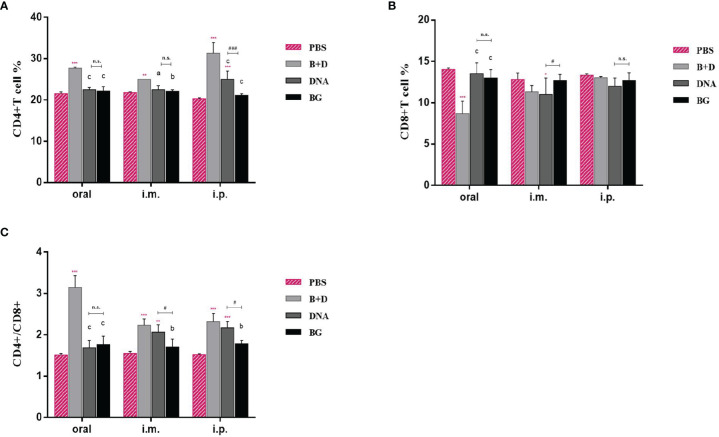
CD4^+^ and CD8^+^ T–lymphocytes populations in immunized mice. At 14 days after the last immunization, the splenocytes of mice from each group were harvested. The percentages of **(A)** CD4^+^ and **(B)** CD8^+^ T cells were analyzed using flow cytometry. **(C)** Proportions of CD4^+^/CD8^+^. Values are expressed as the mean ± SD of four individual experiments. Significance of differences between the PBS, bacterial ghost (BG)+DNA, DNA, and BG groups is represented as follows: ^*^P < 0.05, ^* *^P < 0.01, ^***^P < 0.001. Letter indicates significant differences between BG +DNA, DNA, and BG groups: ^a^P < 0.05, ^b^P < 0.01, ^c^P < 0.001. #Asterisk indicates significant difference between DNA and BG groups: ^#^P < 0.05, ^##^P < 0.01, ^###^P < 0.001. n.s., no significance.

We analyzed the percentages of CD4^+^ and CD8^+^ T cells after isolating splenic lymphocytes from the mice in each group. As shown in **Figure 10**, vaccination with pCI-PoRV-VP6 or pCI-PoRV-VP6-loaded BGs induced changes in the populations of CD4^+^ and CD8^+^ T cells (**Figures 12A, B**). The proportions of both CD4^+^/CD8^+^ T cells from mice administered with DNA or DNA-loaded BGs increased significantly compared to those in the mice administered with PBS or BGs (**Figure 12C**). These results indicate that pCI-PoRV-VP6-loaded BGs promoted the proliferation of T cells and polarized Th cells into CD4^+^ T cells to enhance the immune response.

### Effect of DNA Loaded by Immune BGs on Th1 and Th2 Cytokines

**Figure 13:**
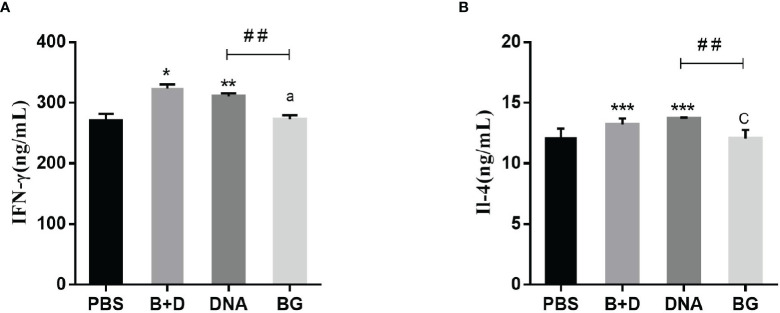
Effect of DNA loaded by immune BGs on Th1 and Th2 cytokines. Serum samples were collected at 24 h after immunization. **(A)** IFN–γ level and **(B)** IL–4 level. Values are expressed as the mean ± SD of four individual experiments. Significance of differences between PBS, BG+DNA, DNA, and BG groups is represented as follows: ^*^P < 0.05, ^**^P < 0.01, ^***^P < 0.001. Letter indicates significant differences between BG +DNA, DNA, and BG group: ^a^P < 0.05, ^b^P < 0.01, ^c^P < 0.001. #Asterisk indicated significant difference between DNA groups and BG groups: ^#^P < 0.05, ^##^P < 0.01, ^###^P < 0.001.

Th1 cytokine (IFN-γ) and Th2 cytokine (IL-4) levels in the serum samples after immunization were evaluated using ELISA. Significant increased levels of IFN-γ were observed after immunization with pCI-PoRV-VP6-loaded BGs compared to in those immunized with PBS in the oral group. Significant levels of IFN-γ were observed after immunization with pCI-PoRV-VP6-loaded BGs compared to those in the immunization with pCI-PoRV-VP6, PBS, or BGs in the intramuscular or intraperitoneal injection groups (**Figure 13A**). Significant levels of IL-4 were observed after immunization with pCI-PoRV-VP6-loaded BGs compared to those following immunization with pCI-PoRV-VP6, PBS, or BGs in the intramuscular injection group (**Figure 13B**). This result indicates that T cells tended to polarize toward the Th1 type.

## Discussion

The use of naked DNA for immunization is a breakthrough in vaccine research. However, DNA possesses poor immunogenicity, and antigens encoded by DNA are not specifically targeted to APCs, limiting its mass implementation. Low efficiency can be achieved by using DNA delivery systems or adjuvants (2). Several studies reported that BGs are effective DNA delivery systems. However, the reported BGs are all made of pathogenic bacteria, typically gram-negative bacteria (11–13), resulting in safety concerns. Moreover, LPS in the cell walls of gram-negative bacteria is associated with the BG envelopes, and it is difficult to eliminate their endotoxic effects (10). We used *L. casei* BGs as a DNA delivery system. Lactic acid bacteria are generally regarded as safe microorganisms. They are widely used in fermentation and are beneficial to animal health (30). The components in the cell walls of lactic acid bacteria can enhance immune responses (11). Moreover, *L. casei* BGs can be mass-produced by fermentation, reducing labor and costs.

Macrophages play important roles in immune processes. They not only fight inflammation and infection, but also trigger innate immune responses. When macrophages are activated, pinocytic activity and phagocytic capacity are increased. DNA-loaded BGs can be efficiently targeted to macrophages, and approximately 60% of cells express the plasmid-encoded reporter gene EGFP (27). Our *in vitro* studies showed that DNA-loaded *L. casei* BGs were efficiently taken up by RAW164.7 macrophages (from 1.4–7.2%) and peritoneal macrophages (from 1.8–57.6%). These results suggest *L. casei* BGs can activate macrophages to enhance their phagocytic capacities and further increase the transfection efficiency of DNA delivery. Macrophages play a role in immune responses by releasing inflammatory factors (nitric oxide) and proinflammatory factors (TNF-α and IL-1β) (31). A major characteristic of polarized and activated macrophages is differential cytokine production. Activated M1 phenotypes of polarized type I responses typically produce IL-12 and TNF-α. In contrast, the M2 phenotypes is associated with IL-10 production (32). Different cytokines integrate into M1 and M2 macrophages to attract T regulatory cells or Th1 and Th2 cells to induce polarized T cell responses. In this study, DNA-loaded *L. casei* BGs significantly upregulated the mRNA expression levels of IL-1β, IL-10, TNF-α, iNOS, Arg-1, and CD206 in RAW264.7 cells and peritoneal macrophages, supporting that *L. casei* BGs effectively activated macrophages, and DNA-loaded *L. casei* BGs upregulated IFN-γ and IL-4 cytokine levels, which are secreted by Th1 and Th2 cells. These results suggest *L. casei* BGs regulate the balance of activated M1 and M2 macrophages and that *L. casei* BGs simultaneously induce Th1- and Th2-type responses.

TLRs are important membrane receptors that activate macrophages. They are engaged by pathogen-associated molecular patterns in cells to activate signaling pathways, driving innate immune functions that initiate adaptive immune responses (33). We found that *L. casei* BGs significantly upregulated the mRNA expression of TLR2 and TLR9 in RAW264.7 cells and peritoneal macrophages. However, the mRNA expression levels of TLR4 were decreased. TLR4 expression in macrophages after stimulation by polysaccharide from the roots of Actinidia (AEPS) has been reported to be similar to our study (34). Additionally, TLR4 expression in RAW264.7 cells and peritoneal macrophages were downregulated by LPS treatment (35, 36). These results suggest that *L. casei* BGs regulate TLRs expression in macrophages, improving the understanding of the immunomodulatory mechanisms of macrophages *via* signaling molecules.

DCs are the most effective APCs and initiate primary immune responses by expressing phenotypic markers and functional cytokines (37). Mature DCs are the only cells that can activate naïve T cells (38). We found that *L. casei* BGs promoted DC maturation and activation. Furthermore, the mRNA expression of IL–6, IL–1β, and IFN–γ was increased in DCs stimulated with DNA–loaded BGs. The mRNA expression of IL–10 and TNF–α were also increased, but it was inapparent compared to those in the DCs incubated with DNA. ELISA assay showed the secretion of the IL–1β, IL–6,IFN–γ IL–10 and TNF–α in DCs were upregulated significantly. IL–1β is a cytokine with many functions and important roles in inducing innate and acquired immune responses (39) {Dinarello, 1996 #20;Dinarello, 1996 #20}. IL–6 is a proinflammatory cytokine (40). TNF–α and IL–10 mainly participate in switching the immunoglobulin class (41). TNF–α also plays an important role in promoting Th2 cells to switch to this class (42). IFN–γ is a key cytokine involved in Th1 polarization and is critical for vaccine–induced protection against pathogens (29). The cytokines released by DCs during activation are important for evaluating stimulated immune responses. *Lactobacillus casei* BGs induced a wide range of chemokines and cytokines in DCs, demonstrating that efficient immune responses can be induced. Mature T cells can be divided into two main subsets, CD4^+^ and CD8^+^, based on their cell–surface identity. The CD4^+^/CD8^+^ ratio is a direct index of immunity (43). In this study, *L. casei* BGs considerably increased the CD4^+^ T cell percentage and CD4^+^/CD8^+^ ratio 14 days after the last immunization. This result suggests that DNA–loaded *L. casei* BGs altered the ratio of T–cell subsets and that humoral immunity and cellular immunity were significantly improved, possibly enhancing immune levels.

BGs contain all surface antigens of bacteria required for activating DCs and macrophages in the immune process (44–46). Therefore, humoral and cell–mediated immune responses are stimulated. The results of our *in vivo* studies showed that DNA–loaded *L. casei* BGs enhanced humoral and, particularly, cell–mediated immune responses. Thus, *L. casei* BGs containing effective immune stimulants may be useful as immunoadjuvants.

## Data Availability Statement

The raw data supporting the conclusions of this article will be made available by the authors, without undue reservation.

## Ethics Statement

The protocol (NEAU2018024) was approved by the Ethical Committee for Animal Experiments of Northeast Agricultural University, China.

## Author Contributions

XLY conducted the Real–time PCR, flow cytometry and ELISA assay. LW drafted the manuscript, conducted the Real-time PCR, flow cytometry and ELISA assay. XRY cultured DCs. SSZ prepared BGs. GL and LZ prepared plasmid. JL and XW immunized the experimental animals. HZ, YJ, and WC analyzed the experimental data. YL, LT, and XQ conceived the project. XQ was the grant holder and drafted the manuscript. All authors read, revised, and approved the final manuscript.

## Funding

This work was supported by grants from the National Natural Science Foundation of China (NSFC) (No. 32072876), Key Research and Development Program of Heilongjiang Province (Grant no: GA21B004), and the Natural Science Foundation of Heilongjiang Province (Grant no: LH2020C022).

## Conflict of Interest

The authors declare that the research was conducted in the absence of any commercial or financial relationships that could be construed as a potential conflict of interest.

## Publisher’s Note

All claims expressed in this article are solely those of the authors and do not necessarily represent those of their affiliated organizations, or those of the publisher, the editors and the reviewers. Any product that may be evaluated in this article, or claim that may be made by its manufacturer, is not guaranteed or endorsed by the publisher.

## References

[1] Brisse M Vrba SM Kirk N Liang Y Ly H. Emerging Concepts and Technologies in Vaccine Development. Front Immunol (2020) 11:583077. doi: 10.3389/fimmu.2020.583077 33101309 PMC7554600

[2] Thanh LN Yin Y Choi Y Jeong JH Kim J. Enhanced Cancer DNA Vaccine *via* Direct Transfection to Host Dendritic Cells Recruited in Injectable Scaffolds. ACS Nano (2020) 14:11623–36. doi: 10.1021/acsnano.0c04188 32808762

[3] Hobernik D Bros M. DNA VaccinesHow Far From Clinical Use? Int J Mol Sci (2018) 19(11):3605. doi: 10.3390/ijms19113605 PMC6274812 30445702

[4] Huter V Szostak MP Gampfer J Prethaler S Wanner G Gabor F. Bacterial Ghosts as Drug Carrier and Targeting Vehicles. J Control Release (1999) 61:51–63. doi: 10.1016/S0168-3659(99)00099-1 10469902

[5] Zhou P Wu H Chen S Bai Q Chen X Chen L. MOMP and MIP DNA–Loaded Bacterial Ghosts Reduce the Severity of Lung Lesions in Mice After Chlamydia Psittaci Respiratory Tract Infection. Immunobiology (2019) 224:739–46. doi: 10.1016/j.imbio.2019.09.002 31561842

[6] Fanuel S Tabesh S Rajani HF Heidari S Sadroddiny E Kardar GA. Decorating and Loading Ghosts With Allergens for Allergen Immunotherapy. Hum Vacc Immunother (2017) 13:2428–33. doi: 10.1080/21645515.2017.1365208 PMC5647983 28934008

[7] Rabea S Alanazi FK Ashour AE Salem–Bekhit MM Yassin AS Moneib NA. Salmonella–Innovative Targeting Carrier: Loading With Doxorubicin for Cancer Treatment. Saudi Pharm J (2020) 28:1253–62. doi: 10.1016/j.jsps.2020.08.016 PMC7584810 33132719

[8] Cai K Tu W Liu Y Li T Wang H. Novel Fusion Antigen Displayed–Bacterial Ghosts Vaccine Candidate Against Infection of Escherichia Coli O157:H7. Sci Rep–Uk (2015) 5:1–10. doi: 10.1038/srep17479 PMC4667225 26626573

[9] Jawale CV Lee JH. Comparative Evaluation of Salmonella Enteritidis Ghost Vaccines With a Commercial Vaccine for Protection Against Internal Egg Contamination With Salmonella. Vaccine (2014) 32:5925–30. doi: 10.1016/j.vaccine.2014.08.072 25218296

[10] Wen J Yang Y Zhao G Tong S Yu H Jin X. Salmonella Typhi Ty21a Bacterial Ghost Vector Augments HIV–1 Gp140 DNA Vaccine–Induced Peripheral and Mucosal Antibody Responses *via* TLR4 Pathway. Vaccine (2012) 30:5733–9. doi: 10.1016/j.vaccine.2012.07.008 22819719

[11] Mader HJ Szostak MP Hensel A Lubitz W Haslberger AG. Endotoxicity Does Not Limit the Use of Bacterial Ghosts as Candidate Vaccines. Vaccine (1997) 15:195–202. doi: 10.1016/S0264-410X(96)00141-7 9066038

[12] Li S Wang D Guo C Tian M Liu Q Pan Z. Study on Preparation of a Streptococcus Suis Ghost Vaccine. Microb Pathog (2021) 154:104865. doi: 10.1016/j.micpath.2021.104865 33771628

[13] Witte A Wanner G Blasi U Halfmann G Szostak M Lubitz W. Endogenous Transmembrane Tunnel Formation Mediated by Phi X174 Lysis Protein E. J Bacteriol (1990) 172:4109–14. doi: 10.1128/jb.172.7.4109-4114.1990 PMC213400 2141836

[14] Kudela P Koller VJ Lubitz W. Bacterial Ghosts (BGs)–Advanced Antigen and Drug Delivery System. Vaccine (2010) 28:5760–7. doi: 10.1016/j.vaccine.2010.06.087 20619379

[15] Cao J Zhang J Ma L Li L Zhang W Li J. Identification of Fish Source Vibrio Alginolyticus and Evaluation of its Bacterial Ghosts Vaccine Immune Effects. Microbiologyopen (2018) 7(3):e576. doi: 10.1002/mbo3.576 PMC6011932 29349911

[16] Thanh LN Yin Y Choi Y Jeong JH Kim J. Enhanced Cancer DNA Vaccine *via* Direct Transfection to Host Dendritic Cells Recruited in Injectable Scaffolds. ACS Nano (2020) 14:11623–36. doi: 10.1021/acsnano.0c04188 32808762

[17] Ji G Zhang Y Si X Yao H Ma S Xu Y. Biopolymer Immune Implants' Sequential Activation of Innate and Adaptive Immunity for Colorectal Cancer Postoperative Immunotherapy. Adv Mater (2021) 33(3):2004559. doi: 10.1002/adma.202004559 33296110

[18] Hafkamp FMJ Groot Kormelink T de Jong EC. Targeting DCs for Tolerance Induction: Don't Lose Sight of the Neutrophils. Front Immunol (2021) 12:732992. doi: 10.3389/fimmu.2021.732992 34675923 PMC8523850

[19] Dieterlen M Klaeske K Bernhardt AA Borger MA Klein S Garbade J. Immune Monitoring Assay for Extracorporeal Photopheresis Treatment Optimization After Heart Transplantation. Front Immunol (2021) 12:676175. doi: 10.3389/fimmu.2021.676175 34447372 PMC8383491

[20] Pires–Lapa MA Koga MM Jr Da Silva IA Filgueiras LR Jancar S. Leukotriene B–4 Modulation of Murine Dendritic Cells Affects Adaptive Immunity. Prostag Oth Lipid M (2019) 141:34–9. doi: 10.1016/j.prostaglandins.2019.02.001 30738873

[21] Batah AM Ahmad TA. The Development of Ghost Vaccines Trials. Expert Rev Vaccines (2020) 19:549–62. doi: 10.1080/14760584.2020.1777862 32500816

[22] Montanaro J Inic–Kanada A Ladurner A Stein E Belij S Bintner N. Escherichia Coli Nissle 1917 Bacterial Ghosts Retain Crucial Surface Properties and Express Chlamydial Antigen: An Imaging Study of a Delivery System for the Ocular Surface. Drug Des Dev Ther (2015) 9:3741–54. doi: 10.2147/DDDT.S84370 PMC4516183 26229437

[23] Hou R Li M Tang T Wang R Li Y Xu Y. Construction of Lactobacillus Casei Ghosts by Holin–Mediated Inactivation and the Potential as a Safe and Effective Vehicle for the Delivery of DNA Vaccines. BMC Microbiol (2018) 18(80):1–9. doi: 10.1186/s12866-018-1216-6 30055567 PMC6064150

[24] Hajam IA Dar PA Appavoo E Kishore S Bhanuprakash V Ganesh K. Bacterial Ghosts of Escherichia Coli Drive Efficient Maturation of Bovine Monocyte–Derived Dendritic Cells. PloS One (2015) 10(12):e0144937. doi: 10.1371/journal.pone.0144397 26669936 PMC4684396

[25] Lee J Park C Jang S Kim J Kim S Song S. Tolerogenic Dendritic Cells are Efficiently Generated Using Minocycline and Dexamethasone. Sci Rep–Uk (2017) 7:15087. doi: 10.1038/s41598-017-15569-1 PMC5678112 29118423

[26] Dar P Kalaivanan R Sied N Mamo B Kishore S Suryanarayana VVS. (TM) 201 Adjuvanted FMD Vaccine Induces Improved Immune Responses and Protection in Cattle. Vaccine (2013) 31:3327–32. doi: 10.1016/j.vaccine.2013.05.078 23735678

[27] Paukner S Kudela P Kohl G Schlapp T Friedrichs S Lubitz W. DNA–Loaded Bacterial Ghosts Efficiently Mediate Reporter Gene Transfer and Expression in Macrophages. Mol Ther (2005) 11:215–23. doi: 10.1016/j.ymthe.2004.09.024 15668133

[28] Hong SJ Kim SK Ko EB Yun C Han SH. Wall Teichoic Acid is an Essential Component of Staphylococcus Aureus for the Induction of Human Dendritic Cell Maturation. Mol Immunol (2017) 81:135–42. doi: 10.1016/j.molimm.2016.12.008 27978487

[29] Scott K Manunta M Germain C Smith P Jones M Mitchell P. Qualitatively Distinct Patterns of Cytokines are Released by Human Dendritic Cells in Response to Different Pathogens. Immunobiology (2005) 116:245–54. doi: 10.1111/j.1365-2567.2005.02218.x PMC1817823 16162273

[30] Sanlier N Gokcen BB Sezgin AC. Health Benefits of Fermented Foods. Crit Rev Food Sci (2019) 59:506–27. doi: 10.1080/10408398.2017.1383355 28945458

[31] Gholijani N Gharagozloo M Farjadian S Amirghofran Z. Modulatory Effects of Thymol and Carvacrol on Inflammatory Transcription Factors in Lipopolysaccharide–Treated Macrophages. J Immunotoxicol (2016) 13:157–64. doi: 10.3109/1547691X.2015.1029145 25812626

[32] Gaiser A Bauer S Ruez S Holzmann K Faendrich M Syrovets T. Serum Amyloid A1 Induces Classically Activated Macrophages: A Role for Enhanced Fibril Formation. Front Immunol (2021) 12:691155. doi: 10.3389/fimmu.2021.691155 34276683 PMC8278318

[33] Pasare C Medzhitov R. Toll–Like Receptors and Acquired Immunity. Semin Immunol (2004) 16:23–6. doi: 10.1016/j.smim.2003.10.006 14751760

[34] Sun H Zhang J Chen F Chen X Zhou Z Wang H. Activation of RAW264.7 Macrophages by the Polysaccharide From the Roots of Actinidia Eriantha and its Molecular Mechanisms. Carbohyd Polym (2015) 121:388–402. doi: 10.1016/j.carbpol.2014.12.023 25659714

[35] Ru Z Xu M Zhu G Tu Y Jiang Y Du H. Ovotransferrin Exerts Bidirectional Immunomodulatory Activities *via* TLR4–Mediated Signal Transduction Pathways in RAW264.7 Cells. Food Sci Nutr (2021) 9:6162–75. doi: 10.1002/fsn3.2569 PMC8565217 34760247

[36] Akashi S Shimazu R Ogata H Nagai Y Takeda K Kimoto M. Cutting Edge: Cell Surface Expression and Lipopolysaccharide Signaling *via* the Toll–Like Receptor 4–MD–2 Complex on Mouse Peritoneal Macrophages. J Immunol (Baltimore Md 1950) (2000) 164:3471–5. doi: 10.4049/jimmunol.164.7.3471 10725698

[37] Sei JJ Ochoa AS Bishop E Barlow JW Golde WT. Phenotypic, Ultra–Structural, and Functional Characterization of Bovine Peripheral Blood Dendritic Cell Subsets. PloS One (2014) 9(10):e109273. doi: 10.1371/journal.pone.0109273 25295753 PMC4190170

[38] Subramanian N Wu Z Reister F Sampaio KL Frascaroli G Cicin–Sain L. Naive T Cells are Activated by Autologous HCMV–Infected Endothelial Cells Through NKG2D and can Control HCMV Transmission *In Vitro* . J Gen Virol (2017) 98:3068–85. doi: 10.1099/jgv.0.000976 29165229

[39] Hong S Yu J. Prolonged Exposure to Lipopolysaccharide Induces NLRP3–Independent Maturation and Secretion of Interleukin (IL)–1 Beta in Macrophages. J Microbiol Biotechnol (2018) 28:115–21. doi: 10.4014/jmb.1709.09017 29061031

[40] Yrlid U Svensson M Johansson C Wick MJ. Salmonella Infection of Bone Marrow–Derived Macrophages and Dendritic Cells: Influence on Antigen Presentation and Initiating an Immune Response. FEMS Immunol Med Microbiol (2000) 27:313–20. doi: 10.1016/S0928-8244(99)00209-6 10727887

[41] Rojas JM Avia M Martin V Sevilla N. IL–10: A Multifunctional Cytokine in Viral Infections. J Immunol Res (2017) 2017:6104054. doi: 10.1155/2017/6104054 28316998 PMC5337865

[42] Choi J Lee S Choi H Kim M Jeon SG Jang MH. House Dust Mite–Derived Chitin Enhances Th2 Cell Response to Inhaled Allergens, Mainly *via* a TNF–Alpha–Dependent Pathway. Allergy Asthma Immun (2016) 8:362–74. doi: 10.4168/aair.2016.8.4.362 PMC4853514 27126730

[43] Stüve O Marra CM Bar–Or A Niino M Cravens PD Cepok S. Altered CD4+/CD8+ T–Cell Ratios in Cerebrospinal Fluid of Natalizumab–Treated Patients With Multiple Sclerosis. Arch Neurol (2006) 63(10 ):1383–7. doi: 10.1001/archneur.63.10.1383 17030653

[44] Hajam IA Dar PA Won G Lee JH. Bacterial Ghosts as Adjuvants: Mechanisms and Potential. Vet Res (2017) 48:1–13. doi: 10.1186/s13567-017-0442-5 28645300 PMC5482964

[45] Senevirathne A Hewawaduge C Park J Park S Lee JH. Parenteral Immunization of Salmonella Typhimurium Ghosts With Surface–Displayed Escherichia Coli Flagellin EnhancesTLR–5 Mediated Activation of Immune Responses That Protect the Chicken Against Salmonella Infection. Microb Pathog (2020) 147:104252. doi: 10.1016/j.micpath.2020.104252 32439565

[46] Peng W Si W Yin L Liu H Yu S Liu S. Salmonella Enteritidis Ghost Vaccine Induces Effective Protection Against Lethal Challenge in Specific–Pathogen–Free Chicks. Immunobiology (2011) 216:558–65. doi: 10.1016/j.imbio.2010.10.001 21247655

