# Supplementary material for: Lactobacillus casei Ghosts as a Vehicle for the Delivery of DNA Vaccines Mediate Immune Responses
Source: Front Immunol. 2022 May 31;13:849409. doi: 10.3389/fimmu.2022.849409 (PMC9193971; doi:10.3389/fimmu.2022.849409)
Supplement: Supplementary file 1 [file Table_1.doc]

# *Lactobacillus casei* ghosts as a vehicle for the delivery of DNA vaccines mediate immune responses

**Xiaoli Yu^1^, Xinru Yang^1^, Songsong Zhang^1^, Guiwei Li^2^, Lanlan Zhang^3^, Jiaxuan Li^1^, Xiaona Wang^1^, Han Zhou^1^,Yanping Jiang^1^, Wen Cui^1^, Yijing Li^1^, Lijie Tang^1^ , Wang Li^1,*^ , Xinyuan Qiao^1,*^**

^1^Heilongjiang Key Laboratory for Animal Disease Control and Pharmaceutical Development, Department of Preventive Veterinary Medicine, College of Veterinary Medicine, Northeast Agricultural University, Harbin, China

^2^Branch of Animal Husbandry and Veterinary of Heilongjiang Academy of Agricultural Sciences, Qiqihar, China

^3^Heilongjiang Fishery Technology extension station, Harbin, China

*** Correspondence:**

Li Wang

wanglicau@163.com

Xinyuan Qiao

qiaoxinyuan@126.com

**Table S1** Primers used in this study.

| Target gene | Primer sequence (5’-3’) |
| --- | --- |
| IL-1β | TGGAGAGTGTGGATCCCAAGCAAT |
|  | TGCTTGTGAGGTGCTGATGTACCA |
| IL-6 | TGGGAAATCGTGGAAATGAG |
|  | CTCTGAAGGACTCTGGCTTTG |
| IL-10 | ACTGCTATGCTGCCTGCTCTTACT |
|  | ACTGGGAAGTGGGTGCAGTTATTG |
| TNF-α | CACAAAACTTGAGAGTCGTGGT |
|  | GCTAGAACCCTAGAGTCAGGC |
| IFN-γ | GCAACAACATAAGCGTCA |
|  | GACCTCAAACTTGGCAATAC |
| iNOS | GTTCTCAGCCCAACAATACAAGA |
|  | GTGGACGGGTCGATGTCAC |
| TLR2 | ATCAGTCCCAAAGTCTAAAGTC |
|  | GGCCAAGTTAGTATCTCTTAGT |
| TLR4 | TTGTATCGCCTTCTTAGCAG |
|  | GGTCCAAGTTGCCGTTTC |
| TLR9 | CCTGCCGCTGACTAATCT |
|  | AAATTGTGGCCTATACCCTTC |
| β-actin | GGCTGTATTCCCCTCCATCG |
|  | CCAGTTGGTAACAATGCCATGT |
| Arg-1 | TGTCCCTAATGACAGCTCCTT |
|  | GCATCCACCCAAATGACACAT |
| CD206 | TGATTACGAGCAGTGGAAGC |
|  | GCTACGACGTGGGCTACAG |
